# 10-Methyldodecanal, a Novel Attractant Pheromone Produced by Males of the South American Cerambycid Beetle *Eburodacrys vittata*

**DOI:** 10.1371/journal.pone.0160727

**Published:** 2016-08-11

**Authors:** Weliton D. Silva, Jocelyn G. Millar, Lawrence M. Hanks, José Maurício S. Bento

**Affiliations:** 1 Department of Entomology and Acarology, University of São Paulo, Piracicaba, São Paulo, Brazil; 2 Department of Entomology, University of California Riverside, Riverside, California, United States of America; 3 Department of Chemistry, University of California Riverside, Riverside, California, United States of America; 4 Department of Entomology, University of Illinois at Urbana-Champaign, Urbana, Illinois, United States of America; Rutgers The State University of New Jersey, UNITED STATES

## Abstract

We report the identification, synthesis, and field bioassay of a novel attractant pheromone produced by males of *Eburodacrys vittata* (Blanchard), a South American cerambycid beetle in the subfamily Cerambycinae. Headspace volatiles from males contained a sex-specific compound, identified as 10-methyldodecanal. In a field bioassay conducted in Brazil, significant numbers of males and females were caught in traps baited with synthesized racemic 10-methyldodecanal, consistent with the aggregation-sex pheromones produced by males of many cerambycine species. This compound represents a new structural class of cerambycid pheromones, and it is the first pheromone identified for a species in the tribe Eburiini.

## Introduction

In the past 12 years, more than 100 pheromones and pheromone candidates have been identified from cerambycid beetle species in the subfamilies Cerambycinae, Lamiinae, Prioninae, Spondylidinae, and Lepturinae, demonstrating that these types of semiochemicals are critical for effective mate location by these beetles (reviewed in [1]). Moreover, increasing evidence suggests that there is considerable parsimony among cerambycid species with regard to pheromone biosynthesis and use, with closely related species sharing pheromone components, or even producing pheromones of apparently identical composition [2–7]. For example, numerous species in the subfamily Cerambycinae produce pheromone components compounds that are 6, 8, or 10 carbons in length, with hydroxyl or carbonyl groups on C2 and C3 [8]. Because of this parsimony, it is common to catch several related species in traps baited with a single compound. Recent work has shown that cross attraction among sympatric cerambycid species is minimized by a number of mechanisms, including differences between species in seasonal and diel activity patterns [9], the use of species-specific pheromone blends rather than single components [9], and vertical stratification of species within the forest canopy [10,11].

Despite the recent rapid progress, it should be noted that pheromones have been identified for less than one percent of the ~35,000 described species within the family [12]. In areas which have been sampled with pheromone-baited traps over a number of years, such as east-central Illinois (see [13]), only a fraction of the endemic species have been caught, suggesting that other pheromone motifs remain to be discovered. Furthermore, most semiochemical research to date has focused on species from North America, Europe, and Asia, with virtually nothing known about the pheromones of species native to Africa, Australia, and South America, despite the diversity of cerambycids on these continents. For example, in Brazil, approximately 4,000 species have been described to date [14], but volatile pheromones used in mate location have been described for only a single species, *Hedypathes betulinus* (Klug) [15]. To begin to fill this knowledge gap, we initiated a research project with the goal of identifying semiochemicals produced by cerambycids native to Brazil. Here, we report some of the first results, for the species *Eburodacrys vittata* (Blanchard) (tribe Eburiini, subfamily Cerambycinae). The known range of this species includes Brazil, Bolivia, Paraguay, Argentina, and Uruguay [16]. In Brazil, larval hosts of *E*. *vittata* include the native “jabuticaba tree”, *Plinia cauliflora* (Mart.) Kausel of the family Myrtaceae [17], and exotic species of *Eucalyptus* of the same family, which were introduced from Australia [18]. In a recent field survey, larvae of this species were found in branches of the native “angico tree”, a species of *Anadenanthera* (Fabaceae) in fragments of the Cerrado or “Brazilian Savanna” (*sensu stricto*, [19]) in Brazil (WDS, personal observation).

As with all other attractant pheromones known from cerambycids in the subfamily Cerambycinae, we found that male *E*. *vittata* produce an aggregation-sex pheromone that attracts both sexes. The structure of the pheromone, 10-methyldodecanal, is entirely different than that of all other cerambycid pheromones reported to date (see [8]), representing the first example of a new structural class of pheromones within this family.

## Materials and Methods

### Source of beetles

We reared adults of *E*. *vittata* from fallen branches of angico trees which were collected weekly from 30 November to 20 December 2014 at a farm in Valentim Gentil, SP, Brazil (20°22'17.7"S, 50°04'46.6"W). This site contains fragments of Cerrado mixed with pastureland. Branches were placed in a nylon net cage (1 × 1 × 2 m) in a shed that was closed on one side, under ambient environmental conditions. Because we had captured adult *E*. *vittata* with light traps in a previous experiment that was intended for other purposes, we inferred that this species was probably nocturnal. Therefore, the cage was checked for emerged adult beetles daily before sunrise for two months. Adult beetles that emerged overnight were sexed by caging them together and observing their mating behavior (i.e., males mount females). The sexes were separated before copulation could take place, and were held separately in 0.2 l plastic containers and shipped overnight to the Laboratory of Chemical Ecology and Insect Behavior at the University of São Paulo, Piracicaba, SP, Brazil. There, beetles were allowed at least 48 h to acclimate under laboratory conditions (23±2°C, 60±10% RH, 12:12 h L:D) prior to being used for collection of headspace volatiles. Beetles were provided 10% sucrose solution in glass tubes (7.5 cm long × 1 cm id) plugged with cotton rolls, as nourishment.

### Identification of the male-specific compound

Volatile compounds produced by adult males and females of *E*. *vittata* were collected by aerating beetles individually in custom made 0.5-liter cylindrical glass chambers that contained a tube with 10% sucrose solution for nourishment. Collectors consisted of glass pipettes (8.5 cm long × 0.5 cm id) containing 150 mg of 80/100 mesh HayeSep^®^ Q (Supelco, Bellefonte, PA, USA) held in place with glass wool plugs. The collector was attached to the outlet of the chamber with a teflon screw cap. Charcoal-purified air was passed through the chamber and collectors at ~300 ml/min. Volatiles were collected continuously for 48 h under the environmental conditions described above, and beetles were aerated as many as three times each. Volatiles were collected from eight adults of each sex. Aerations of chambers without beetles were run simultaneously as controls to check for system contaminants. Trapped volatiles were eluted from collectors with four 0.25-ml aliquots of methylene chloride into silanized glass vials that were stored at -30°C until analysis.

Aeration extracts were initially analyzed by gas chromatography-flame ionization detection (GC/FID) with a Shimadzu GC-2010 gas chromatograph (Shimadzu Corporation, Kyoto, Japan) fitted with an HP5-MS capillary column (30 m × 0.25 mm i.d. × 0.25 μm film; Agilent Scientific, Santa Clara, CA, USA). Injections (2 μl aliquots) were made in splitless mode with an injector temperature of 250°C and helium as carrier gas. The GC oven was programmed from 35°C for 1 min, increased to 40°C at 2°C/min (hold for 1 min), and then increased to 250°C at 10°C/min (hold for 10 min). Extracts that contained sex-specific peaks were shipped to JGM at the University of California, Riverside. These extracts were reanalyzed in splitless mode with an Agilent 7820A GC coupled to a 5977E mass selective detector, using an HP-5 column, and a temperature program of 40°C/1 min, 10°C/min to 280°C, hold for 10 min.

Two of the headspace extracts of male-produced volatiles contained essentially only the single compound in estimated quantities of several micrograms per extract. Consequently, the two samples were combined and concentrated almost to dryness under a slow stream of nitrogen, 100 μl of CD_2_Cl_2_ was added and the sample was concentrated down again, a second 100 μl of CD_2_Cl_2_ was added followed by concentration to ~10 μl, and the remaining sample was transferred to a microbore NMR tube. Proton NMR spectra were taken on a 600 MHz Bruker Avance spectrometer.

### Synthesis of racemic 10-methyldodecanal 7

All reactions using air-or water-sensitive reagents were carried out in oven-dried glassware, under argon atmosphere. Tetrahydrofuran was freshly distilled from benzophenone-sodium ketyl before use. Solutions were dried over anhydrous Na_2_SO_4_ and concentrated by rotary evaporation under reduced pressure. NMR spectra were taken on a Varian Inova 400 instrument, as CDCl_3_ or CD_2_Cl_2_ solutions.

Dihydropyran (7.56 g, 90 mmol) was added to an ice-cooled solution of 1-octynol **1** (10.0 g, 79 mmol) and 100 mg *p*-toluenesulphonic acid (PTSA) in 100 ml ether. After stirring 10 min, the mixture was warmed to room temperature and stirred 4 h. The mixture was then poured into 100 ml of 1 M NaHCO_3_ and shaken vigorously, relieving the gas pressure frequently. The ether layer was then removed and washed with brine, dried, concentrated, and Kugelrohr distilled, giving 15.87 g (96%) of the protected alcohol **2**, bp ~100°C at 0.4 mm Hg.

Trifluoromethanesulphonic anhydride (17.6 g, 80 mmol) was added to a solution of racemic 2-methylbutan-1-ol (6.16 g, 70 mmol) and pyridine (5.6 ml, 71 mmol) in 100 ml dry CH_2_Cl_2_, cooled in an ice-acetone bath to ~-15°C. The cooling bath was then removed and the mixture was warmed to room temperature and stirred 1 h, then diluted with 200 ml pentane. After stirring 10 min, the slurry was filtered through a plug of silica gel pre-wetted with pentane, rinsing with a 2 × 50 ml CH_2_Cl_2_/pentane, 1:2. The solution was concentrated and the crude triflate **3** was used immediately in the next step.

Butyllithium (2.65 M in hexanes) was added slowly to an ice-cooled solution of alkyne **2** (15.5 g, 74 mmol) and triphenylmethane indicator (~50 mg) in dry THF until the solution turned pink, indicating an excess of BuLi. A solution of the crude triflate **3** in 25 ml THF was then added dropwise, keeping the internal temperature <-5°C. After the addition was complete, the mixture was allowed to warm to room temperature overnight, then quenched with saturated aqueous NH_4_Cl. The mixture was extracted with hexane, and the hexane layer was washed with saturated aqueous NaHCO_3_ and brine, then dried and concentrated to give crude alkyne **4**. Crude **4** was taken up in 100 ml MeOH, 100 mg PTSA was added, and the mixture was stirred 2 h at room temperature. Solid NaHCO_3_ (1 g) was then added, and most of the MeOH was removed by rotary evaporation. The residue was then diluted with water and extracted with hexane, and the hexane layer was washed with brine, then dried and treated with decolorizing charcoal. After filtering and concentration, the residue was purified by Kugelrohr distillation, removing a forerun containing THP-protected MeOH (oven at room temp, 0.12 mm Hg). The product was then distilled at ~ 90°C at 0.05 mm Hg, yielding alkynol **5** (12.79 g) contaminated with about 10% 1-octynol **1**. EIMS (70 eV) (*m/z*, abundance): 181 (M+-15, 3), 149 (3), 122 (10), 121 (14), 110 (31), 107 (34), 95 (77), 93 (66), 91 (35), 82 (42), 81 (81), 80 (33), 79 (80), 77 (35), 67 (65), 57 (73), 55 (55), 43 (34), 41 (100). ^1^H NMR (CDCl_3_): δ 3.61 (t, 2H, J = 6.64 Hz), 2.12 (m, 2H), 2.09 (ddt, 1H, J = 16.3, 5.7. 2.3 Hz), 1.98 (ddt, 1H, J = 16.3, 6.8, 2.3 Hz), 1.55–1.3 (m, 8H), 1.17 (m, 1H), 0.91 (d, 3H, J = 6.6 Hz), 0.84 (t, 3H, J = 7.4 Hz). ^13^C NMR (CDCl_3_): δ 81.0, 79.3, 63.1, 34.7, 32.8, 29.3, 28.8, 28.7, 25.9, 25.4, 19.3, 18.9, 11.7 ppm.

Alcohol **5** was taken up in 100 ml hexane, 0.5 g 5% Pd on C was added, and the mixture was stirred under a hydrogen atmosphere for 3 h. The mixture was then filtered through a plug of celite, rinsing with hexane, then Kugelrohr distilled, yielding alcohol **6** (11.9 g, bp <100°C at 0.15 mm Hg) as a colorless oil. EIMS (70 eV) (*m/z*, abundance): 182 (M+-18, trace), 153 (8), 125 (22), 111 (24), 97 (78), 83 (82), 70 (91), 69 (84), 57 (69), 55 (100), 43 (47), 41 (81). ^1^H NMR (CDCl_3_): δ 3.60 (t, 2 H, J = 6.6 Hz), 1.52 (quint, 2H, J ~ 7 Hz), 1.50 (br s, OH), 1.37–1.17 (m, 15H), 1.08 (m, 2H), 0.82 (t, 3H, J = 7.2 Hz), 0.81 (d, 3H, J = 6.4 Hz). ^13^C NMR (CDCl_3_): δ 63.2, 36.8, 34.6, 33.0, 30.2, 29.82 (2C), 29.68, 29.64, 27.28, 25.9, 19.4, 11.6 ppm.

A solution of oxalyl chloride (3.2 ml, 37.5 mmol) in anhydrous CH_2_Cl_2_ (100 ml) was cooled to -78°C under Ar. A solution of dimethylsulphoxide (5.3 ml, 75 mmol) in anhydrous CH_2_Cl_2_ (25 ml) was added dropwise. After 30 min, a solution of 10-methyldodecanol **6** (5.01 g, 25 mmol) in anhydrous CH_2_Cl_2_ (25 ml) was added and the mixture was stirred for 1 h. Et_3_N (13.9 ml, 100 mmol) then was added and stirring continued for 30 min. The cold bath was removed and the mixture was stirred for 1.5 h while warming to room temperature. The mixture was poured into water, and extracted with CH_2_Cl_2_. The combined organic layers were concentrated. The residue was taken up in hexanes and washed with 1 M HCl, saturated aqueous NaHCO_3_, and brine, then dried and concentrated. The residue was purified by Kugelrohr distillation (discarding the forerun at 60°C / 0.4 mm Hg containing octanal and MeOTHP). The product was distilled at <100°C / 0.1 Torr, yielding 2.94 g of **7** as a yellow liquid (GC purity 92%). EIMS (70 eV) (*m/z*, abundance): 198 (M+, trace), 180 (1), 169 (5), 151 (20), 125 (16), 123 (21), 109 (40), 95 (93), 83 (56), 82 (59), 81 (61), 71 (47), 70 (81), 69 (59), 67 (46), 57 (100), 55 (76), 43 (54), 41 (82). ^1^H NMR (CD_2_Cl_2_): δ 9.72 (t, 1H, J = 1.8 Hz), 2.39 (td, 2H, J = 7.4, 1.8 Hz), 1.58 (m, 2H), 1.21–1.35 (m, 14H), 1.15–1.05 (m, 2H), 0.85 (t, 3H, J = 7.1 Hz), 0.83 (d, 3H, J = 6.3 Hz). ^13^C NMR (CD_2_Cl_2_): δ 202.9, 44.0, 36.8, 34.6, 30.1, 29.7, 29.5, 29.3, 27.2, 24.9, 22.3, 19.1, 11.3 ppm.

### Field bioassay of synthesized pheromone

Synthetic racemic 10-methyldodecanal was tested in a field bioassay conducted in Valentim Gentil (see above) from 5 October 2015 to 15 January 2016. We used custom-built panel traps (black corrugated plastic) modeled after those used by Mitchell et al. [9] that were hung from inverted L-shaped hangers constructed of polyvinyl chloride irrigation pipe (for details, see [20]). The trap surfaces were coated with Fluon^®^ diluted 1:1 with water (Insect-a-Slip, BioQuip Products Inc., Rancho Dominguez, CA, USA) to increase trapping efficiency [20]. The bottoms of the trap basins (5-l plastic jars) were filled with a few cm of water to which were added a few drops of detergent and one teaspoon of NaCl as a preservative.

Lures consisted of clear low-density polyethylene press-seal bags (Bagettes^®^ model 14770, 5 × 7.5 cm, 50 μ wall thickness; Cousin Corp., Largo, FL, USA) loaded with 50 mg of racemic 10-methyldodecanal in 950 μl of isopropanol. Control lures consisted of bags loaded with 1 ml of isopropanol. Lures were hung with wire in the open central slot of the panel traps. Traps were placed ~15 m apart in four pairs which were ~30 m apart. Pheromone and control treatments were assigned randomly to traps, and each pair included one treatment and one control trap. Traps were serviced every 4–5 d, at which time treatments within trap pairs were switched to control for positional effects. Lures were replaced every two weeks.

Differences between treatments in mean trap catch of adults of *E*. *vittata* (sexes combined), blocked by collection date, were tested with the nonparametric Friedman’s test (PROC FREQ with CMH option; [21]) because assumptions of analysis of variance were violated by heteroscedasticity [22]. The sex ratio of captured beetles was compared to a nominal 1:1 ratio with the χ2 goodness-of-fit-test. Replicates (block × collection date) in which no beetles were caught in any of the traps in that block of traps were excluded from the analyses, leaving 39 replicates in total. Voucher specimens have been deposited in the collection of the entomology museum in the Department of Entomology and Acarology, University of São Paulo, Piracicaba, SP, Brazil.

Collection of beetles and field bioassays were conducted under SISBIO permit # 46395–1 and # 46395–2 from the Brazilian Ministry of the Environment. Permission for accessing the field sites in Valentim Gentil was granted by the property owner, Cassio Dias da Silva (to WDS).

## Results

### Identification and synthesis of the male-specific compound

**Fig 1:**
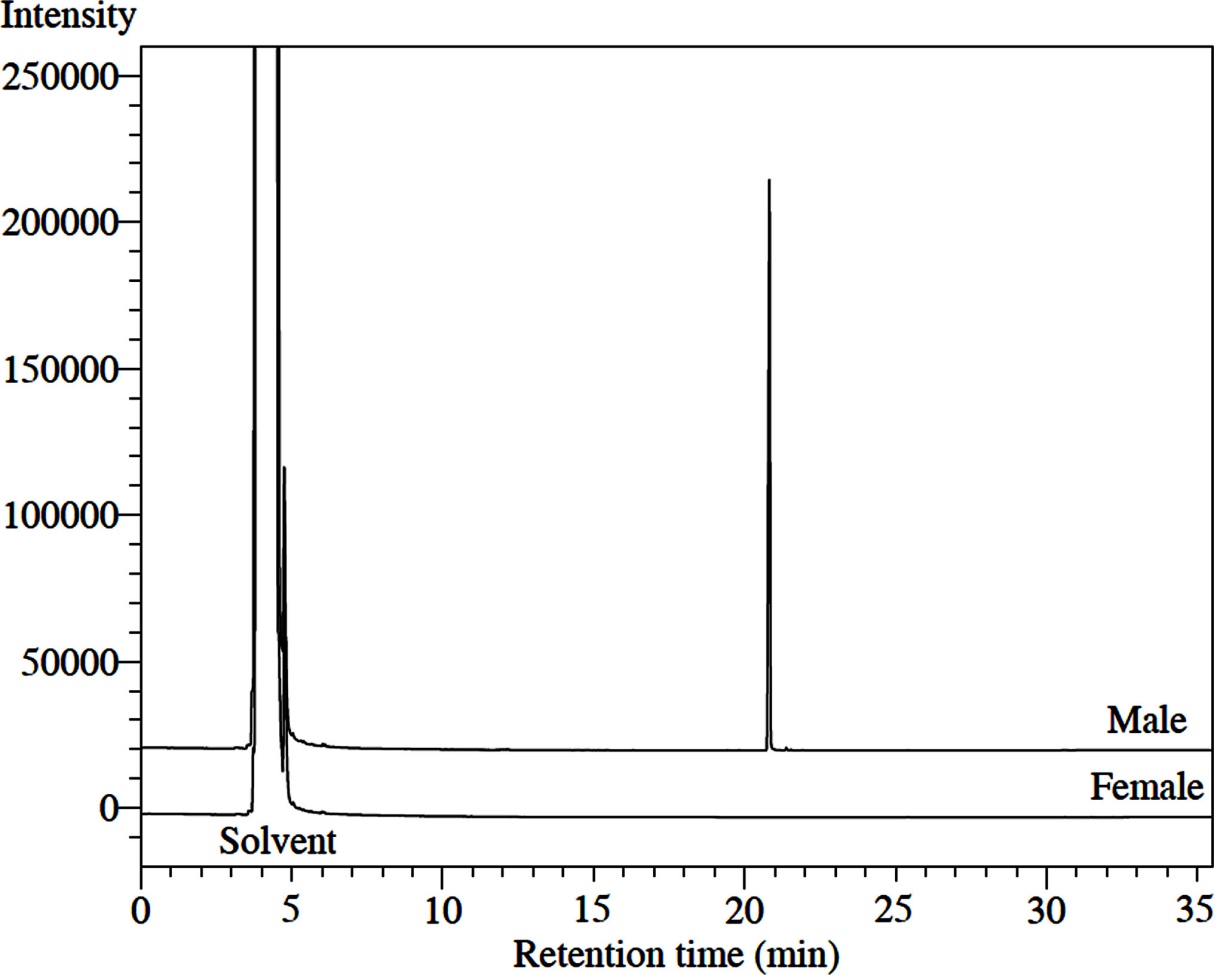
Gas chromatograms of extracts of volatiles produced by adult males and females of *Eburodacrys vittata*, showing the single male-specific compound.

**Fig 2:**
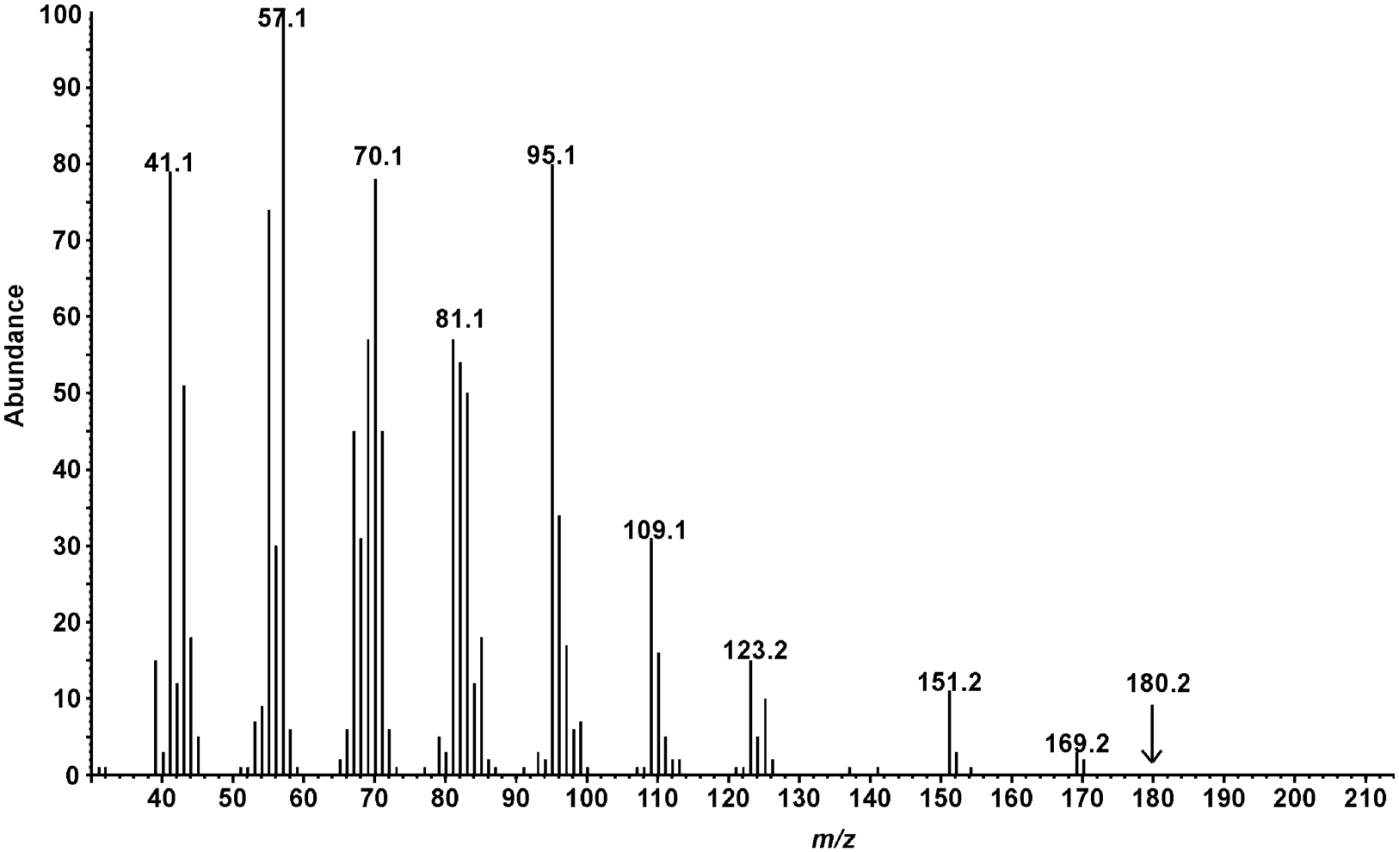
EI mass spectrum of male-specific *Eburodacrys vittata* compound.

Aeration extracts from male beetles were very clean, consisting of only a single sex-specific compound (Fig 1). The EI mass spectrum of this compound (Fig 2) showed a trace ion at *m/z* 198, and an ion at *m/z* 180 from loss of water, suggesting that the molecular ion was indeed 198, for a possible molecular formulae of C_13_H_26_O with one site of unsaturation, or C_12_H_22_O_2_, with two sites of unsaturation. The retention indices of 1491 on a nonpolar HP-5 column and 1595 on a medium polarity DB17 column favored the former, with one heteroatom and a single site of unsaturation.

The proton NMR spectrum of the insect-produced compound showed an aldehyde proton at 9.73 ppm (t, J = 1.8 Hz), coupled to a methylene at 2.39 ppm (td, J = 7.4, 1.8 Hz), which was in turn coupled to a methylene at 1.60 ppm (m). All other protons had chemical shifts less than 1.5 ppm, ruling out the possibility of any other heteroatoms or sites of unsaturation, confirming the molecular formula as C_13_H_26_O. The remainder of the spectrum consisted of a methylene multiplet at ~1.2–1.35 ppm (14 H), a one-proton multiplet at 1.12 ppm, a terminal methyl group at 0.85 ppm (t, J = 7.2 Hz), and a methyl group at 0.83 ppm (d, J = 6.3 Hz) attached to a methine carbon, i.e., a methyl group somewhere along a carbon chain. Thus, the data supported an X-methyldodecanal structure, where X could not be positions 2 or 3, which were clearly methylenes, or position 11, which would have resulted in an isopropyl group rather than a terminal methyl and an internal methyl. Thus, the methyl group could be on any one of carbons 4–10.

**Fig 3:**
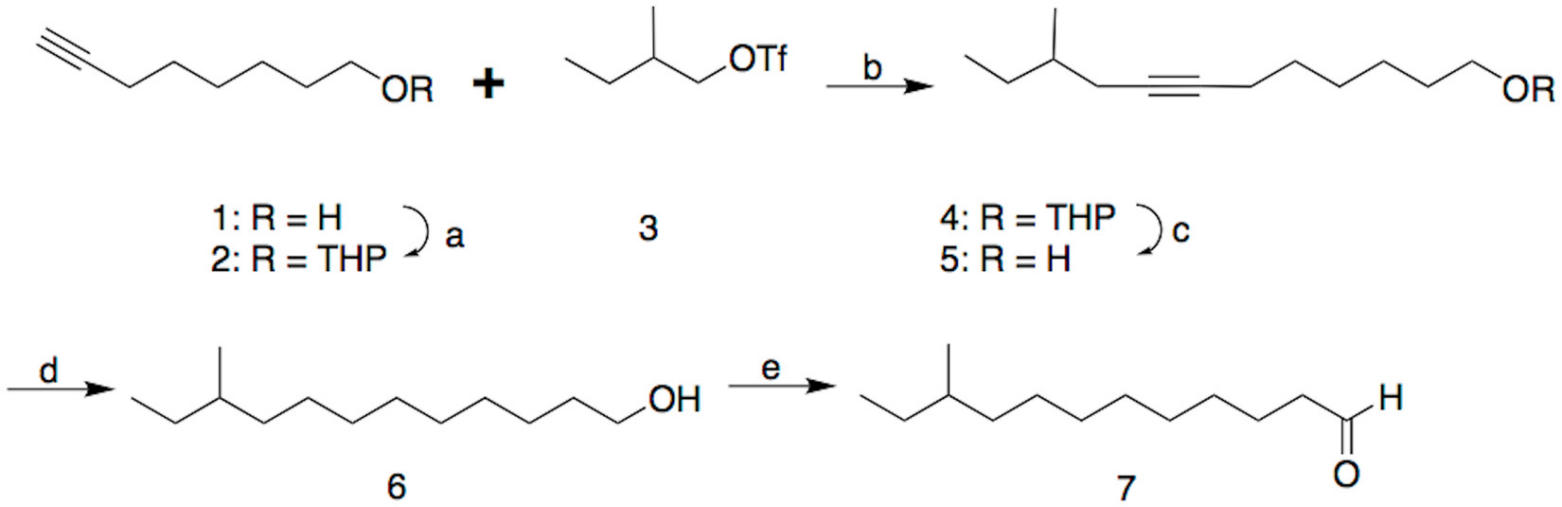
Synthesis of 10-methyldodecanal. a) Dihydropyran, PTSA, ether; b) BuLi, THF; c) MeOH, PTSA; d) 5% Pd on carbon, H_2_, hexane; e) Pyridinium dichromate, CH_2_Cl_2_.

Careful reexamination of the mass spectrum suggested 10-methyldodecanal. Specifically, the first significant ions occurred at *m/z* 169 (5%, M+-29) and 151 (20%, M+-18 and 29), suggesting favored loss of an ethyl group from the parent molecule at a branch point, or the parent molecule after loss of water. Furthermore, most of the mass spectrum was characterized by regularly spaced clusters of ions about 14 mass units apart, as would be expected from fragmentations along a chain of methylenes. However, there was a marked gap in the spectrum between *m/z* 123 and 151, corresponding to 28 instead of 14 mass units, as would occur with cleavage on either side of a methyl branch point on C10, after initial loss of water from the parent molecule. From these data, the molecule was tentatively identified as 10-methyldodecanal, and this was confirmed by synthesis of an authentic standard in several straightforward steps (Fig 3).

Thus, the alcohol function of 7-octyn-1-ol **1** was protected as the THP derivative. The protected alkynol **2** was deprotonated with butyllithium, and the resulting alkynyl anion was reacted with racemic 2-methylbutan-1-ol triflate **3** to give **4**. Acid-catalyzed removal of the THP protecting group, Pd-catalyzed hydrogenation of the alkyne in **5**, and pyridinium dichromate oxidation of the resulting alcohol **6** gave the desired aldehyde **7** as the racemate. Neither alcohol **6** nor aldehyde **7** were resolved on a chiral stationary phase Cyclodex B GC column, and so it was not possible to determine which enantiomer of aldehyde **7** the beetles produced.

### Field bioassay of the synthesized pheromone

Traps baited with racemic 10-methyldodecanal captured 155 adults of *E*. *vittata* (56 males and 99 females; sex ratio significantly different from 1:1, *χ*2 = 11.9, *P* = 0.0006). Baited traps caught a mean (±1 SE) of 4.0 ± 0.44 beetles per replicate, whereas no beetles were caught in control traps (Friedman’s Q_1,78_ = 66.3, *P* < 0.0001). In addition, baited traps caught 2 males and 6 females of the congener *Eburodacrys fortunata* Lameere and 2 males and 1 female of *Susuacanga octoguttata* (Bates) (also in the tribe Eburiini), whereas no beetles of either species were caught in control traps. No other species of insects were captured in consistent or significant numbers by either the pheromone-baited or the control traps.

## Discussion

As with many other cerambycids in the subfamily Cerambycinae [1], males of *E*. *vittata* emitted comparatively large quantities (micrograms per male) of a single pheromone compound. Attraction of both sexes to traps baited with synthetic 10-methyldodecanal indicated that this compound is an aggregation-sex pheromone (*sensu* [23]), as appears to be true for pheromones produced by all other species in the subfamily Cerambycinae for which pheromones have been identified, as well as species in the subfamilies Lamiinae and Spondylidinae (tabulated in [1]). Moreover, 10-methyldodecanal represents the first example of a new and unexpected pheromone structure for cerambycid beetles, being entirely different from any of the previously known structures. It was not possible to determine which enantiomer the insects produce because the enantiomers of either the aldehyde **7** or its immediate precursor, alcohol **6**, were not resolved on a chiral stationary phase GC column. Thus, the absolute configuration of the insect-produced compound will have to determined indirectly by synthesis and field testing of the two enantiomers.

Although the sex ratio of the adults of *E*. *vittata* that were reared from larval hosts was approximately 1:1, our trap catches were female biased (≈1.8:1). However, without some independent measure of the actual sex ratio in the field during the course of the bioassays, it is impossible to determine whether or not this reflects females being more strongly attracted to 10-methyldodecanal than males.

There also was evidence that 10-methyldodecanal may represent a new pheromone motif that is shared among closely related species, as suggested by the attraction of males and females of the congener *E*. *fortunata*, and of a third species in the same tribe, *S*. *octoguttata*, to traps baited with 10-methyldodecanal. Many pheromone structures are known to be highly conserved within the Cerambycidae, with congeners and even more distantly related species commonly sharing pheromone components, even among species on different continents which have been separated for millions of years (e.g., [2–7]). However, to confirm conservation of pheromone structures among the species in the present study, it will be necessary to collect headspace volatiles from both sexes of *E*. *fortunata* and *S*. *octoguttata*, and verify that males of these species do indeed produce 10-methyldodecanal.

Given that *E*. *vittata* has been reported as a pest of *Eucalyptus* species in Brazil [18], traps baited with synthetic 10-methyldodecanal could become useful tools for management. Pheromone-based sampling also could be used to delineate the current geographic range of this poorly known species, as well as being useful for detecting introductions outside of its native range as an invasive species, especially in the many other parts of the world where eucalypts are grown in commercial plantations or as landscape trees.

In summary, this work extends our knowledge of the chemical ecology of the Cerambycidae, especially of species native to South America. It is particularly important because the field bioassays in which species from two genera within the tribe Eburiini were caught, suggest that the pheromone component, 10-methyldodecanal, may be the first representative of a new pheromone motif, rather than a structure unique to a single species. Given that there are approximately 255 described species within the tribe Eburiini [24], it seems likely that a number of these species may use 10-methyldodecanal or related compounds as pheromones.

## Supporting Information

S1 File Spreadsheet containing the number of adult *Eburodacrys vittata* captured by traps baited with racemic 10-methyldodecanal. (XLSX)
